# Professional Stakeholders' Perspectives on Early Screening for Type 1 Diabetes in Children: A Systematic Review

**DOI:** 10.1002/dmrr.70240

**Published:** 2026-10-19

**Authors:** Francesca Gatti, Bianca M. Milazzo, Lauren M. Quinn, Antonio Catarinella, Gaia Perego, Francesca Milano, Karin Lange, Sheila Greenfield, Renuka Dias, Jurgen Vercauteren, Adya Rao, Aymeric Mahieu, Carmen Hurtado del Pozo, Valentina E. Di Mattei, Katharine Barnard, Chantal Mathieu, Parth Narendran, Emanuele Bosi, Olga Boiko, EDENT1FI, Chantal Mathieu, Lut Overbergh, Pieter Gillard, Kristina Casteels, Jurgen Vercauteren, Anette Ziegler, Peter Achenbach, Christiane Winkler, Sandra Hummel, Florian Haupt, Jose Maria Zapardiel Gonzalo, Thomas Pieber, Julia Mader, Bart Torbeyns, Zdenek Sumnik, Ondrej Cinek, Vit Neuman, Barbora Berka, Flemming Pociot, Julie Christine Antvorskow, Julie Hviid Klaebel, Riitta Lahesmaa, Heikki Hyöty, Jutta Laiho, Felix Reschke, Olga Kordonouri, Thekla von dem Berge, Ezio Bonifacio, Angela Hommel, Gita Gemulla, Anja Loff, Emanuele Bosi, Lorenzo Piemonti, Vito Lampasona, Francesco Dotta, Guido Sebastiani, Agnieszka Szypowska, Lidia Groele, Przemyslawa Jarosz‐Chobot, João Filipe Raposo, Rogerio Ribeiro, Raquel Coelho, Mark Peakman, Elisabeth Neiemoeller, Jessica Dunne, Charlie Nicholls, Aymeric Mahieu, Iker Mille Irure, Jan Liska, Philippe Bordes, Luciana Gusukuma, Regine Bergholdt, Dorte Blaume Boldsen, Matthias Von Herrath, Ohad Cohen, Simona de Portu, Maria Ida Buompensiere, Carmen Hurtado del Pozo, Esther Latres, Sanjoy Dutta, Gina Agiostratidou, Anne Koralova, James Jackson, Hugo Tewson, David Darrock, Nagalla Srinivasa, Joseph Hedrick, Kimberly Collins, Tim Tree, Colin Dayan, Rebecca Playle, Peter Taylor, Rachel E. J. Besser, Parth Narendran, Ian Litchfield, Sheila Greenfield, Olga Boiko, Renuka Dias, Shu Chen, Marie Amoroso, Katherine Barnard‐Kelly, Daniel Agardh

**Affiliations:** ^1^ School of Psychology Vita‐Salute San Raffaele University Milan Italy; ^2^ Diabetes and Endocrinology Department General Medicine IRCCS San Raffaele Scientific Institute Milan Italy; ^3^ Institute of Immunology and Immunotherapy College of Medicine and Health University of Birmingham Birmingham UK; ^4^ Department of Psychology University of Milan‐Bicocca Milan Italy; ^5^ Clinical and Health Psychology Unit IRCCS San Raffaele Scientific Institute Milan Italy; ^6^ Department of Medical Psychology Hannover Medical School Hannover Germany; ^7^ Department of Applied Health Research College of Medicine and Health University of Birmingham Birmingham UK; ^8^ Department of Paediatric Endocrinology Birmingham Children's Hospital Birmingham UK; ^9^ Department of Chronic Diseases and Metabolism KU Leuven Leuven Belgium; ^10^ Health Economics and Value Assessment Department Sanofi Paris Île‐de‐France France; ^11^ Research Department Breakthrough T1D New York New York USA; ^12^ Spotlight‐AQ Limited Portcheste Hampshire UK; ^13^ Department of Endocrinology University Hospitals Leuven Leuven Belgium; ^14^ School of Medicine Vita‐Salute San Raffaele University Milan Italy

**Keywords:** presymptomatic type 1 diabetes, professional stakeholders, systematic review, T1D screening programme

## Abstract

Early screening for type 1 diabetes (T1D) is receiving increasing attention as presymptomatic screening initiatives and disease‐modifying therapies continue to expand. However, the perspectives of professional stakeholders, who play a pivotal role in implementation, patient engagement, and health system preparedness, have not been systematically synthesised. We conducted a systematic review to examine professional views on paediatric early T1D screening. Following PRISMA guidelines, six databases (Medline, Embase, Scopus, CINAHL, PsycINFO, ASSIA) and grey literature were searched through January 2026. Twelve studies met the inclusion criteria, predominantly involving paediatricians and diabetes specialists; nurses, genetic counsellors, policymakers, and other healthcare professionals were represented in few studies. Methodological appraisal using the Mixed Methods Appraisal Tool (MMAT) identified recurring limitations in sample representativeness and potential nonresponse bias. Given the heterogeneity and primarily descriptive nature of the study design and outcomes, findings were synthesised narratively. Professional perspectives varied according to the population targeted, professional group studied, screening scenario, and the implementation context. Where directly compared, support was greater for at‐risk than for general‐population screening. Reported benefits included diabetic ketoacidosis (DKA) prevention, earlier clinical engagement, and improved preparedness for families. However, concerns persisted regarding psychosocial burden, workforce capacity, and unclear referral and follow‐up pathways. More favourable evaluations were reported in some structured programmes, although these findings may reflect reliance on existing infrastructures and targeted participant selection. By consolidating previously fragmented evidence, this review identifies recurring clinical, psychosocial, and organisational considerations relevant to paediatric T1D screening. These findings primarily reflect clinical perspectives and indicate conditional rather than uniform support. Further studies should evaluate how standardised pathways, professional training, and integrated psychosocial support affect the feasibility and acceptability of screening across different healthcare contexts.

## Graphical abstract

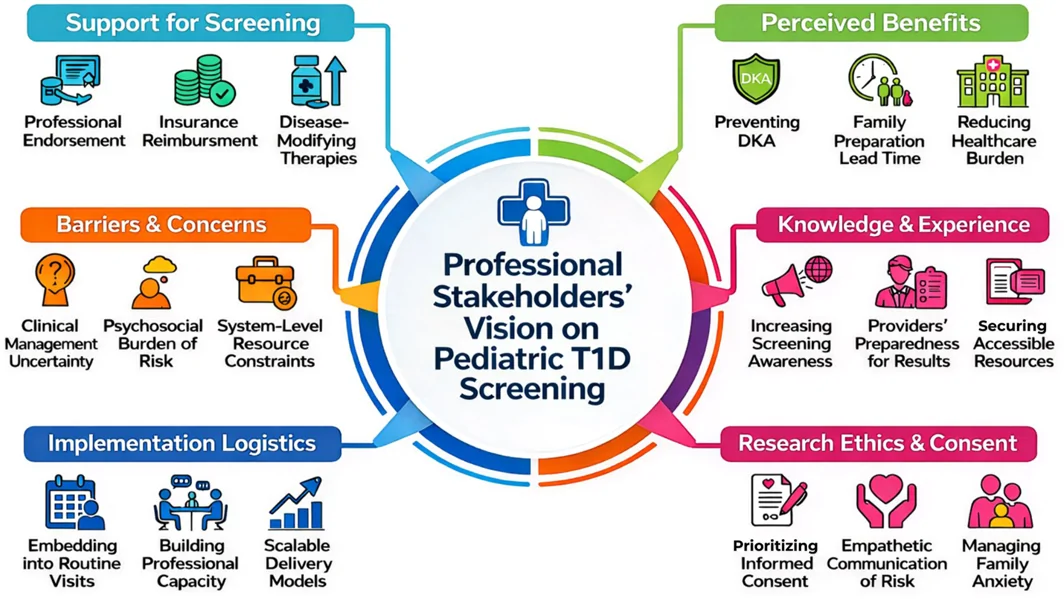

## 1 Introduction

Type 1 diabetes (T1D) is a chronic autoimmune disease characterised by progressive destruction of pancreatic β‐cells, resulting in insulin deficiency [1]. In Europe, it affects over 400,000 children and adolescents, with an annual incidence of approximately 15 per 100,000 individuals [2]. T1D may remain undetected for a prolonged period, progressing silently until insulin deficiency becomes clinically evident [3]. The disease develops through three stages: two presymptomatic phases characterised by circulating islet autoantibodies (Stage 1: associated with normoglycemia; Stage 2: associated with dysglycemia), followed by Stage 3, defined by overt hyperglycemia, clinical symptoms, and the need to start insulin therapy [4]. At diagnosis, many paediatric patients present with diabetic ketoacidosis (DKA), a potentially life‐threatening complication, with reported rates ranging from 13% to 80% across countries [5]. Beyond acute complications, T1D substantially affects long‐term morbidity, mortality, and quality of life for patients and their families [6, 7, 8, 9].

Early screening for T1D aims to identify Stage 1 or Stage 2 disease before symptomatic onset, either in the general paediatric population or in children at increased risk, particularly those with a first‐degree relative with T1D or an identified genetic susceptibility. Screening approaches include genetic risk assessment, islet autoantibody testing (IAb), and combined or sequential strategies integrating both methods. Early identification enables structured monitoring and may reduce the risk of DKA at diagnosis [10, 11]. Its clinical relevance has increased with the development of disease‐modifying therapies. Teplizumab is the first treatment approved by the U.S. Food and Drug Administration for Stage 2 T1D and more recently recommended for NHS use in the UK, can delay progression to Stage 3 by approximately 3 years on average [12]. Longitudinal initiatives such as TEDDY, launched in 2004 [13], established early frameworks for presymptomatic identification, while population‐based programmes such as Fr1da, introduced in Germany in 2015, examined the integration of screening into routine paediatric care [14].

Population‐based screening research is currently being investigated in several countries. The European EDENT1FI programme (www.edent1fi.eu), for example, evaluates the feasibility, acceptability, and longer‐term implications of paediatric T1D screening across centres in Germany, the Czech Republic, Denmark, Sweden, Italy, Poland, Portugal, and the United Kingdom [15].

As these programmes expand, their implementation depends not only on scientific evidence and therapeutic developments, but also on the perspectives and preparedness of the professional stakeholders involved in their organisation and delivery. Professional perspectives are relevant because screening delivery, communication, referral, and follow‐up depend on the knowledge, attitudes, and operational capacity of those involved [16]. Evidence from paediatric HIV testing interventions suggests that professional stakeholder engagement is a critical determinant of responsible implementation and clinical uptake [17]. However, despite the growing number of screening initiatives, no systematic review has synthesised the perspectives of professional stakeholders towards early T1D screening in children.

Against this background, the present systematic review examined how professional stakeholders perceive early T1D screening in children. For this review, professional stakeholders were defined as healthcare professionals and other professional or service‐level actors involved in the design, governance, organisation, delivery, communication, or follow‐up of screening. These included primary care clinicians, diabetes specialists, nurses, genetics professionals, psychologists, policymakers, administrators, and insurers. We aimed to capture the range of these perspectives and explore how they varied across the targeted population, screening approach, professional group, geographical setting, and implementation context.

## 2 Methods

The review was conducted and reported in accordance with the PRISMA 2020 statement; no substantial deviations from these guidelines occurred. The protocol was registered on PROSPERO (CRD42024566937).

### 2.1 Search Strategy

We searched six electronic databases (Medline (OVID), EMBASE (OVID), Scopus, Cumulative Index to Nursing and Allied Health Literature (CINAHL), PsycINFO, and Applied Social Sciences Index and Abstracts (ASSIA)) until April 2025, supplemented by an updated search conducted in January 2026 to capture studies published in 2025–2026 (the Medline search strategy is available in Table S1). Grey literature, including articles' reference lists, conference abstracts, and additional electronic searches, was also performed.

No restrictions were applied regarding language, country, study design, or publication year. In addition, the reference lists of relevant reviews and included articles were screened to identify further studies.

### 2.2 Article Selection

We imported citations into Covidence, which removed duplicates. Titles and abstracts were independently screened (FG/BMM/AC/AR) and disagreements were resolved by team discussion. Reviewers applied pre‐specified criteria to determine eligibility (Table S2). Disagreements in full‐text articles were resolved by a moderator (GP). Two reviewers independently performed data extraction (FG/BMM).

We included studies that tested children (< 18 years) for T1D via genetic, IAb, or combined approaches. Study designs included prospective cohorts, observational studies, nonrandomised studies, and cross‐sectional studies, adopting quantitative, qualitative, or mixed methodologies. We examined a broad range of professional stakeholders' perspectives—including views, attitudes, beliefs, knowledge, experiences, practices, and preferences. In this review, the term ‘professional stakeholders’ refers to healthcare professionals and other professional actors involved in paediatric T1D care or screening governance, including paediatricians, diabetologists, nurses, genetic counsellors, policymakers, health system managers, and related professionals.

### 2.3 Quality Assessment

Methodological quality was assessed using the Mixed Methods Appraisal Tool (MMAT) [18, 19]. Design‐specific criteria were applied according to whether the studies were quantitative descriptive, qualitative, or mixed methods, with each criterion rated as ‘Yes’, ‘No’, or ‘Can't tell’. Two reviewers (FG and BMM) independently performed the assessments.

### 2.4 Data Synthesis

Quantitative findings were synthesised descriptively, preserving the original constructs, denominators, response categories, and screening scenarios. Qualitative findings and open‐text responses were extracted as author‐reported themes, subthemes, or participant accounts. All findings were organised in a study‐by‐finding evidence matrix recording the stakeholder group, geographical setting, screening target and approach, and implementation context.

The organisation and presentation of findings were informed by the thematic synthesis approach described by Thomas and Harden [20], adapted for a body of quantitative, qualitative, and mixed‐methods evidence. Recurring concepts were compared across studies and grouped into six higher‐order thematic domains. Each finding was assigned to one primary thematic domain to minimise duplication, while differences according to geographical and healthcare setting, professional group, target population, screening approach, implementation experience, and therapeutic context were examined descriptively. Because the studies addressed different questions and used different measures and response formats, their findings were not combined statistically and formal subgroup analyses were not undertaken.

## 3 Results

### 3.1 Literature Search and Study Characteristics

The study selection process is illustrated in the PRISMA flow diagram (Figure 1). The database search yielded 12,476 records (Embase, *n* = 6959; Medline, *n* = 3900; Scopus, *n* = 1071; CINAHL, *n* = 304; PsycINFO, *n* = 200; ASSIA, *n* = 42). Grey literature, including articles' reference lists, conference abstracts, and additional electronic searches, was also performed. These studies (*n* = 4) were assessed against the same inclusion criteria as those retrieved from the database search.

**FIGURE 1:**
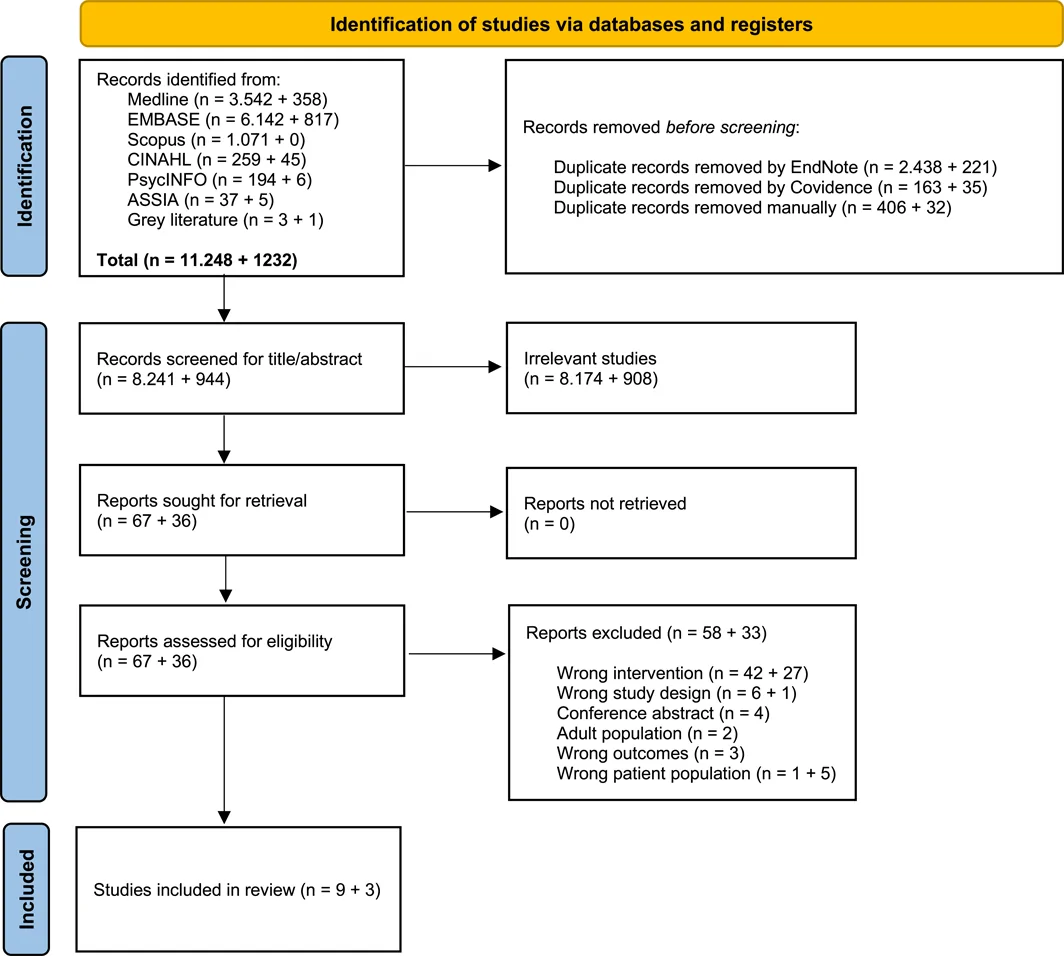
PRISMA flow diagram. Numbers reported correspond to the original database search (April 2025); additional records identified through the updated search conducted in January 2026, covering studies published in 2025–2026, are indicated using the ‘+’ symbol.

Following deduplication in EndNote (*n* = 2659), Covidence (*n* = 198), and manual checking (*n* = 438), 9185 unique records remained. Title and abstract screening excluded 9082 records, leaving 103 articles for full‐text assessment. All included studies were published in English. Of the 103 full texts assessed, 91 were excluded from not meeting the inclusion criteria, resulting in a total of 12 studies included in the review. Across the 12 included studies, a total of 2125 professional stakeholders contributed data to the review, with study‐level samples ranging from 25 to 500 participants.

Study and sample characteristics are summarised in Table S3. Of the 12 included studies, eight were quantitative descriptive, two qualitative, and two mixed methods. Most studies recruited paediatricians, primary care professionals, endocrinologists, or other healthcare professionals directly involved in clinical care. One study focused exclusively on genetic counsellors, while nonclinical stakeholders, including policymakers, insurers, and community or service‐level actors, were represented in only two studies and generally in small subsamples. Most studies examined hypothetical or pre‐implementation screening scenarios, whereas direct programme‐based or research‐informed experience was concentrated in a limited number of studies. Consequently, the findings primarily reflect the perspectives of healthcare professionals directly involved in clinical care rather than those of the broader range of stakeholders involved in screening governance, organisation, and implementation.

### 3.2 Quality Assessment

Quantitative descriptive, qualitative, and mixed‐methods studies were appraised using the corresponding MMAT criteria. The qualitative studies met all applicable criteria. Among the quantitative descriptive studies, recurring limitations concerned sample representativeness and potential nonresponse bias. In the mixed‐methods studies, some uncertainty concerned the interpretation of integrated findings and the handling of potential divergences between quantitative and qualitative components. Study‐level appraisal findings are reported in Table S4.

### 3.3 Sources of Variation Across Studies

The included studies varied in geographical setting, professional group, target population, screening modality, outcome definition, degree of implementation experience, and therapeutic context, limiting direct comparisons across studies. Seven studies were conducted in the United States, whereas the remaining studies were conducted in Germany, Italy, the United Kingdom, and Australia, and one survey study included respondents from 76 countries [21, 22, 23, 24, 25, 26, 27, 28, 29, 30, 31, 32]. The three earliest U.S. studies focused primarily on hypothetical genetic susceptibility testing, newborn‐screening policy, and consent [21, 22, 23]. In contrast, subsequent studies more often addressed islet autoantibody screening, monitoring, costs, clinic workflows, and follow‐up arrangements.

Studies also differed in whether they focused on children at increased risk or the general paediatric population; where both groups were directly compared, support was greater for at‐risk screening [21, 22, 23, 24]. Primary care studies generally focused on preparedness and integration into routine care, whereas specialist studies more often examined result communication, monitoring, referral, and follow‐up. However, these patterns arose from separate studies using different measures and did not constitute direct comparisons between professional groups. Variation in outcome definitions and response formats precluded the calculation of a single overall estimate. Implementation contexts ranged from purely hypothetical screening scenarios to studies conducted in preparation for planned programmes and settings in which professionals had direct experience of research or established screening initiatives. Such experience was reported mainly in Fr1da and in selected specialist, international, and research settings [24, 26, 28, 32]. Studies were conducted both before and after the regulatory approval of teplizumab, although approval did not necessarily correspond to local availability or access. Because the therapeutic period varied alongside geographical setting, screening approach, target population, and implementation context, its specific association with professional perspectives could not be assessed separately.

None of the included studies prespecified a threshold at which anticipated clinical benefit, psychosocial burden, treatment‐related risk, workload or cost would make screening acceptable or unacceptable; these outcomes were therefore retained as study‐specific findings and were not synthesised.

### 3.4 Findings by Themes

The synthesis identified six higher‐order themes: support for screening; perceived benefits; concerns and barriers; knowledge and clinical experience; implementation logistics; and research ethics and consent (Table S5). Findings within each theme are presented below, retaining the constructs and screening contexts examined in the original studies.

#### 3.4.1 Theme 1—Support for Screening

##### 3.4.1.1 Variation in Support by Screening Target and Implementation Context

In early U.S. surveys, support differed according to the target population and screening scenario. Among paediatricians, 34% supported testing high‐risk infants, compared with 9% for universal newborn screening and 23% for population screening beyond the newborn period [21]. Among genetic counsellors, 20% supported testing high‐risk infants, 6% supported newborn screening and 23% supported later population screening [22]. A further U.S. survey found that 41% of paediatricians supported testing high‐risk infants and 25% (55/223) supported universal newborn screening [23]. In an international survey, 81.7% (339/415) of healthcare professionals supported screening the at‐risk paediatric population, whereas 26.2% (102/389) supported general‐population screening; for the latter, 35.7% (139/389) were opposed, and 38.0% (148/389) were undecided [24].

Other studies assessed support‐related outcomes within specific implementation contexts. In the implemented Fr1da programme, 90% of participating primary care paediatricians and all nine responding diabetes care centres rated screening as medically useful [28]. In Italy, 84% of 62 family paediatricians reported willingness to participate in the planned national screening programme [27]. In a U.S. primary care survey involving clinicians with limited direct screening experience, 75% of 106 paediatric primary care providers indicated that they were likely to offer screening in the future [25]. Among 50 U.S. endocrinology providers, 52% reported strong clinic‐level interest and support for autoantibody screening and a further 30% reported mild interest and support. Support was more selective by target group: first‐degree relatives (90%), individuals with multiple autoimmune diseases (64%), and those with elevated glucose levels (62%) were most frequently identified as likely to benefit, whereas 60% considered the general population least likely to benefit; 52% were cautious about recommending general‐population screening and 28% did not recommend it [26]. In a U.S. pre‐implementation study examining the proposed integration of screening into well‐child visits, 66.7% of 15 primary care clinicians approved of screening and 73.3% found it appealing [31].

##### 3.4.1.2 Determinants Shaping Support

Among U.S. paediatric primary care providers, factors rated as very important in the decision to offer screening included a first‐degree family history of T1D (77%), parental request (51%), and the presence of other autoimmune conditions (50%) [25]. For example, support for universal newborn screening was higher among paediatricians practising in states where T1D newborn‐screening research activities were present than among those practising in other states (36% vs. Twenty‐one percent) [23].

In a U.S. discrete choice experiment, 94% of paediatricians reported being likely or very likely to offer screening if their preferred test were available, recommended by the American Academy of Paediatrics, and reimbursed by insurance [29]. In UK qualitative, hypothetical interviews, stakeholders supported further research into general‐population screening but generally did not support its immediate routine implementation; most indicated that the availability of a licenced preventive therapy would increase their support [30].

#### 3.4.2 Theme 2—Perceived Benefits

##### 3.4.2.1 Clinical Benefits

Prevention of diabetic ketoacidosis (DKA) has been identified as a benefit across several studies [24, 25, 26, 27, 29, 30]. Among 106 U.S. paediatric primary care providers, 93% selected DKA prevention and 88% the opportunity to intervene to delay disease progression; other reported benefits included earlier insulin initiation (52%), avoidance of misdiagnosis as type 2 diabetes (43%), and referral to research studies (41%) [25]. Among 50 U.S. endocrinology providers, 90% identified early diabetes management or intervention as a benefit, followed by research enrolment (84%), clinical guidance and diagnostic clarity (80%), and prevention of DKA and related hospitalisation (76%) [26]. In Italy, where multiple responses were permitted, 95% of 62 family paediatricians identified DKA reduction as a benefit and 27% identified the potential to delay disease progression [27]. In qualitative interviews with 25 UK professional stakeholders, DKA prevention was described as a priority, and a few participants considered education and monitoring to reduce DKA sufficient in themselves to justify general‐population screening [30]. In a discrete‐choice experiment involving 500 U.S. paediatricians, changing from education alone, with an assumed DKA risk of 15%–20%, to monitoring every 3 months, with an assumed risk of ≤ 1%, was one of the two most influential screening attributes, together with avoiding a $50 out‐of‐pocket cost [29]. These DKA‐risk estimates were attributes of the hypothetical screening scenarios rather than clinical outcomes measured in the study.

##### 3.4.2.2 Family‐Related Benefits

Among Italian family paediatricians, 44% identified that screening could give children and families more time to adjust to the diagnosis [27]. In UK qualitative interviews, participants identified family education, earlier symptom recognition, monitoring, preparation for a future diagnosis, and a smoother transition to Stage 3 T1D as potential benefits. Earlier identification was also described as facilitating access to diabetes technology and supporting glycaemic management following clinical diagnosis [30].

##### 3.4.2.3 Organisational and System‐Level Benefits

Among the nine participating diabetes care centres, most reported lower family burden and reduced initial care workload compared with symptomatic diagnosis. Seven centres rated family burden as lower, six reported less work for initial education, and seven reported less work for initial treatment. For long‐term care, five centres reported a lower workload and four reported a similar workload [28].

#### 3.4.3 Theme 3—Perceived Barriers and Concerns

##### 3.4.3.1 Clinical Concerns

In early U.S. surveys, open‐text responses from paediatricians and genetic counsellors highlighted the absence of preventive treatment, uncertain predictive value and genotype–phenotype associations, potential parental confusion or anxiety, false reassurance, and concerns about testing being used primarily for research [21, 22]. As these concerns emerged from open‐text responses rather than prespecified survey items, they were reported qualitatively and no prevalence estimates were available.

More recent surveys examined uncertainty regarding screening and subsequent management. Among 50 U.S. endocrinology providers, 46% identified uncertainty about the treatment plan following a positive result as a barrier; within the paediatrician‐subgroup, 47% selected the absence of an effective therapy [26]. In Italy, 53% of 62 family paediatricians were concerned about the absence of an available preventive treatment as a concern, while 31% reported the need for repeated autoantibody testing over time [27]. Among 106 U.S. paediatric primary care providers, not knowing how to interpret screening tests was rated as a significant barrier by 50%, and not knowing which tests to order by 45% [25].

##### 3.4.3.2 Family‐Related and Psychosocial Concerns

Among U.S. endocrinology providers, 70% identified anxiety or stress among patients and families as a barrier [26]. In Italy, 86% of family paediatricians were concerned that screening could increase family anxiety, while 69% expressed concern about identifying children who might not progress to clinical T1D [27]. Among U.S. paediatric primary care providers, psychosocial impact was rated as a significant barrier by 11% and as a somewhat significant barrier by 51%; limited availability of psychosocial resources was rated as significant by 27% and somewhat significant by 53% [25].

In UK qualitative interviews, stakeholders described the burden of uncertainty regarding the timing of progression to Stage 3 T1D, changes in family dynamics, stigma, and the medicalisation of an otherwise asymptomatic child. Concerns also included premature health anxiety and possible effects on the child's right to an ‘open future’ [30]. Australian healthcare professionals described difficulties communicating prognostic uncertainty to families and uncertainty about their professional role in providing emotional support or referring families for specialist psychological care [32].

##### 3.4.3.3 Organisational and System‐Level Barriers

Among U.S. endocrinology providers, 66% identified screening costs and 54% limited insurance coverage as barriers. Clinic‐level limitations included insufficient time (60%), understaffing (26%), and limited resources (22%); 16% reported no established workflow for communicating results, while 22% reported that their clinic did not provide follow‐up education or support programmes [26]. UK stakeholders reported limited staffing, funding, resources, and healthcare‐system capacity, together with uncertainty regarding the cost‐effectiveness of population screening and preventive interventions [30]. In U.S. interviews, clinicians described time and staffing constraints, difficulties obtaining paediatric blood samples, and limited preparedness to interpret results, while health insurers identified absent or uncertain insurance coverage as a barrier [31]. Italian family paediatricians also reported workload and time constraints and uncertainty regarding follow‐up procedures [27].

#### 3.4.4 Theme 4—Knowledge and Clinical Experience

##### 3.4.4.1 Awareness and Knowledge

Among 62 Italian family paediatricians, 24% reported knowing nothing and 52% knowing only a little about the national screening programme, while 26% were unaware of their expected role in REF. Correct knowledge of progression risk was reported by 37% for Stage 1% and 47% for Stage 2 T1D. Although 76% correctly identified the general paediatric population as the screening target, 13% selected only relatives of people with T1D and 11% selected families with autoimmune conditions [27]. In a U.S. survey published in 2025, 30% of 106 paediatric primary care providers were familiar with T1D staging, 34% with research screening programmes, and approximately 5% had previously screened a patient [25]. In an earlier U.S. survey published in 2006, 42% (93/223) of paediatricians were unsure or did not answer whether experimental T1D newborn screening was available in their state [23].

##### 3.4.4.2 Preparedness and Confidence

In the Italian survey, 73% of family paediatricians did not feel adequately prepared to fulfil their expected role in the screening programme. Nevertheless, 56% expressed confidence in managing the follow‐up of children identified as positive within their own practices, while the remaining respondents indicated that ongoing monitoring should be conducted by regional paediatric diabetes teams. Paediatricians reporting greater confidence were older than those reporting lower confidence (median age 53.0 vs. 47.0 years, *p* = 0.048) [27]. Among U.S. paediatric primary care providers, only 25% felt comfortable ordering screening tests and 20% interpreted the results. Reported comfort was higher for reviewing diabetes symptoms (87%), providing lifestyle counselling (90%), discussing diabetes risk (66%), and assessing glucose tolerance in autoantibody‐positive children (61%) [25].

Among 50 U.S. endocrinology providers, 96% felt confident describing available screening tests and 94% felt confident sharing and discussing results with patients and families [26]. In the implemented Fr1da programme, 145 of 148 primary care paediatricians (97%) and all paediatric diabetes care centres rated the information received from the coordinating centre as good or very good [28]. These Fr1da items assessed satisfaction with programme‐provided information rather than factual knowledge or clinical competence.

##### 3.4.4.3 Educational Needs

In Italy, all family paediatricians expressed interest in receiving further theoretical and practical information about the screening programme, and 75% indicated a need for additional training and support [27]. In Australian interviews with 32 healthcare professionals, participants described developing knowledge primarily through self‐directed, experiential, and peer‐based learning, while reporting limited formal education. They requested further guidance on screening and monitoring procedures, progression risk associated with different autoantibody profiles, continuous glucose monitoring (CGM) use, insulin initiation, and clinical trial availability. They also sought guidance on communicating risk and uncertainty to families and providing emotional support within professional boundaries [32].

UK stakeholders similarly identified the need for clear information and management pathways to guide referral and follow‐up after screening, particularly for primary care professionals who might be approached by families following a positive result [30]. In a U.S. mixed‐methods study, clinicians and health insurers identified education for healthcare professionals and families as a facilitator of screening implementation [31].

#### 3.4.5 Theme 5—Implementation Logistics

##### 3.4.5.1 Screening Delivery

In the implemented Fr1da programme, 69% of primary care paediatricians rated the integration of screening into daily practice as good or very good, while 70% considered its permanent integration into standard check‐ups very or somewhat useful [28]. Among 62 Italian family paediatricians, 95% reported that they would introduce screening during routine health check‐ups [27]. Suggested approaches to introducing screening included posters in paediatric practices (53%), brochures (34%), telephone contact (16%), and email (8%); some respondents also proposed discussing screening during influenza vaccinations or other consultations. While 58% were familiar with the health visitor role, 94% believed that health visitors could play an important role in programme promotion and implementation [27].

UK stakeholders proposed delivering screening through general practice, schools, and existing vaccination appointments, and emphasised the importance of convenient and easy‐to‐use tests [30]. In a discrete‐choice experiment involving 500 U.S. paediatricians, respondents generally preferred tests that avoided venipuncture and could be performed on the same day at their office or preferred laboratory. Within the range of hypothetical attributes presented, however, the mode, location, and timing of test administration had less influence on preferences than education and monitoring arrangements and families' out‐of‐pocket screening costs [29].

##### 3.4.5.2 Organisational Capacity

In Fr1da, the availability of the coordinating centre and the services it provided were rated as good or very good by 92% and 98% of primary care paediatricians, respectively. All paediatric diabetes care centres rated their collaboration with and the availability of the coordinating centre as good or very good [28].

In a U.S. mixed‐methods study, 11/14 paediatric primary care clinicians with complete feasibility data (78.6%) rated screening during well‐child visits as implementable, possible, and doable, while 9/14 (64.3%) considered it easy to use. Interviews with 10 clinicians and two health insurers identified integration with other scheduled blood tests, electronic health record tools, clinic protocols, institutional policies and leadership, and insurance coverage or incentives as facilitators [31].

In Australian interviews with 32 healthcare professionals, participants raised questions about healthcare‐system capacity to manage increased numbers of children identified through screening, including the need for additional clinics and psychological support, CGM integration, and clearer delineation of professional roles. They proposed nationally consistent, profession‐tailored education and a community of practice focused on early‐stage T1D clinics [32].

##### 3.4.5.3 Follow‐Up Pathways

In Fr1da, among 49 primary care paediatricians who had cared for at least one child identified through screening, 40/49 (82%) rated the support provided by paediatric diabetes care centres to children and families as good or very good [28].

Among 50 U.S. endocrinology providers, 74% reported that screening was available within their facility or hospital system, 34% referred patients to external screening programmes and 28% ordered tests through external laboratories. When asked how results were or would be communicated, 52% selected telephone, 48% a patient portal, and 46% a dedicated appointment. Forty‐two percent reported that their clinic provided follow‐up education or support programmes for families after result disclosure, whereas 22% reported no such support and 28% were unsure [26].

In the international survey, screening was available at 210/431 respondents' centres (48.7%) for at‐risk children and 87/358 (24.3%) for the general paediatric population. Most respondents did not currently follow screen‐positive children but reported willingness to do so if the opportunity and infrastructure were available [24]. Among U.S. paediatric primary care providers, HbA1c (96%) and random glucose testing (90%) were the most frequently selected monitoring methods. The preferred point of referral to paediatric endocrinology varied: before autoantibody testing (22%), for result interpretation (25%), for subsequent monitoring (24%), or after abnormal glucose tolerance was identified (29%) [25]. UK stakeholders also proposed registries for at‐risk children, recording screening results in medical records, and clear referral and monitoring pathways [30].

#### 3.4.6 Theme 6—Research Ethics and Consent

##### 3.4.6.1 Consent Preferences

In early U.S. surveys, consent preferences varied according to the screening scenario presented. Among paediatricians supporting population screening later in infancy, most favoured voluntary participation with informed consent, although a T1D‐specific numerator and denominator were not reported [21]. Among genetic counsellors supporting later population screening for T1D, 94% (58/62) preferred voluntary screening and 6% (4/62) mandatory screening [22]. Among the 55 paediatricians who supported universal newborn screening for T1D, the authors reported that almost half favoured requiring parental consent, but did not provide the exact number. Consent preferences did not significantly differ between physicians practising in states with and without T1D newborn‐screening research activities [23].

In UK qualitative interviews, stakeholders generally favoured a voluntary opt‐in approach and considered the acceptability of screening less established among general‐population families without previous experience of T1D than among families with a first‐degree relative [30].

##### 3.4.6.2 Research Participation and Preventive Interventions

In the UK qualitative study, 64% (16/25) of stakeholders supported further research into paediatric general‐population T1D screening despite the absence of a licenced preventive treatment, while a further 24% (6/25) indicated that they would support it if a preventive treatment such as teplizumab were licenced in the UK [30]. Participants also raised concerns about immunotherapies, including uncertain long‐term adverse effects and the suitability of infusion‐based treatments for children. One paediatric diabetes consultant noted that only a minority of eligible children with a first‐degree relative had enroled in prevention studies; this observation was not quantified across the sample [30].

In the international survey, 66.2% (284/429) of healthcare professionals were willing to participate in secondary prevention trials involving at‐risk children and 65.2% (208/319) in trials involving children identified through general‐population screening [24].

##### 3.4.6.3 Communicating Uncertainty

Australian healthcare professionals described difficulties communicating detectable autoantibodies and uncertain progression timelines to their families. Participants emphasised the use of simple and consistent language, empathy, and communication tailored to families' individual circumstances. They also reported uncertainty about their role in providing emotional support [32].

##### 3.4.6.4 Child‐ and Family‐Related Ethical Concerns

In UK qualitative interviews, stakeholders raised concerns about labelling and medicalising asymptomatic children, including stigma, premature health anxiety, loss of childhood innocence, and possible threats to the child's right to an ‘open future’. Psychological counselling and peer support were identified as possible components of the support offered to families living with risk [30]. In the Italian survey, some family paediatricians also questioned whether the resources required for screening should instead be allocated to other healthcare priorities [27].

## 4 Discussion

This systematic review synthesised quantitative and qualitative evidence on professional perspectives regarding early screening for T1D in children. The available evidence did not identify a uniform level of professional support across settings. The clearest finding from studies directly comparing screening targets was greater support for screening children at increased risk, including first‐degree relatives, than for general‐population screening [21, 22, 23, 24]. Support for broader population screening was more variable and was frequently conditional on the availability of professional guidance, reimbursement, preventive options, and clearly defined referral and follow‐up pathways. Prevention of diabetic ketoacidosis (DKA), earlier clinical engagement, and greater time for family preparation were the most frequently reported benefits, whereas prognostic uncertainty, psychosocial burden, workforce capacity, and unclear clinical responsibilities were recurrent concerns.

These findings should be interpreted in light of substantial contextual and methodological variations across studies. The earlier studies were conducted in the United States and examined hypothetical genetic susceptibility testing, newborn‐screening policy, and consent before current T1D staging frameworks and disease‐modifying therapies were available [21, 22, 23]. Later, studies more often examined islet autoantibody screening within research, pilot, pre‐implementation, or established programme settings. The study period was therefore closely associated with differences in screening modality, target population, geographical setting, professional composition, implementation experience, and therapeutic context. Similarly, the more favourable evaluations reported in some tailored programme‐based settings may reflect the availability of established infrastructures and the inclusion of professionals already engaged in screening, rather than an independent effect of implementation experience [28, 31, 32].

The stronger support observed for at‐risk than for general‐population screening may partly reflect the higher baseline risk in these children and the resulting clearer clinical rationale for testing and follow‐up. Professionals may also anticipate greater familiarity with T1D among families with an affected relative, making screening and risk communication appear more acceptable or manageable. Findings from families with a history of T1D should therefore not be extrapolated to general‐population screening, particularly because studies also differed in screening methods, consent procedures, follow‐up arrangements, and therapeutic context.

Direct geographical comparisons were not possible because studies conducted in different countries also differed in professional samples, screening approaches, study period, and implementation experience. Nevertheless, the issues examined reflected their healthcare and implementation contexts. U.S. studies more frequently addressed insurance coverage, reimbursement, out‐of‐pocket costs, and clinic‐level workflows. The German study evaluated an implemented and centrally coordinated programme, whereas the Italian and UK studies examined pre‐implementation or proposed pathways within publicly organised healthcare systems; the Australian study focused on professionals with experience of research screening and early‐stage monitoring. Professional context also shaped the focus of the studies: primary care studies more often addressed familiarity, preparedness, and integration into routine care, while specialist studies more frequently examined result communication, monitoring, referral, and follow‐up. However, because primary care and specialist professionals were examined in separate studies using different samples and measures, the available evidence does not permit direct comparisons of professional competence. These patterns should therefore be interpreted as contextual differences across studies rather than as demonstrated country or professional‐group effects. The evidence base was also predominantly clinical. Most participants were paediatricians, primary care professionals, endocrinologists, or other healthcare professionals directly involved in clinical care. Policymakers, administrators, insurers, school professionals, and other actors involved in programme governance and resource allocation were represented in only a small number of studies and usually in limited subsamples.

Prevention of DKA was the most consistently identified clinical benefit although the included studies primarily assessed perceived or anticipated benefits rather than clinical outcomes following implementation. Professionals also valued earlier access to monitoring and care, opportunities for preventive research or treatment, and greater time for families to prepare for a future diagnosis. However, no study established the degree of DKA reduction, delay in disease progression, or improvement in family preparedness that professionals would consider sufficient to justify general‐population screening.

Implementation‐related findings concerned the organisation of care following screening as much as the screening procedure itself. Professionals identified the need for clear protocols, defined professional roles, accessible specialist referral, structured monitoring pathways, adequate staffing, reimbursement, and education addressing both clinical and communication‐related aspects of screening. Programme‐based evidence from Fr1da indicated that primary care paediatricians and diabetes care centres valued access to a coordinating centre and structured specialist support [28]. However, because these findings were not derived from controlled comparisons, they do not establish that coordinated infrastructures independently increase professional support or improve implementation outcomes. Recent guidance similarly emphasises coordinated follow‐up, shared‐care arrangements, and psychosocial support, although the effectiveness of different delivery models requires direct comparative evaluation [33, 34]. Emerging glucose‐monitoring technologies may provide additional options for longitudinal surveillance. Rossi et al. evaluated the clinical performance of a novel system in individuals with established T1D; however, its applicability to antibody‐positive children and presymptomatic monitoring remain to be evaluated [35].

Practical implementation resources may help operationalise some of the needs identified in this review. For example, the BR1DGE programme has developed a T1D Early Detection Toolkit addressing screening and confirmatory testing, communication of results, monitoring, and referral pathways [36]. However, this resource was not evaluated in the studies included in the review, and its effectiveness, transferability across healthcare systems, and impact on professional preparedness or screening outcomes remain to be established.

Finally, concerns about psychosocial burden were reported across professional groups and settings. These included family anxiety, uncertainty regarding progression, stigma, medicalisation of an asymptomatic child, and the possible effects of risk knowledge on family relationships and the child's future self‐perception. Such concerns were often anticipated by professionals rather than measured directly in participating families. Evidence from a separate mixed‐methods systematic review suggests that early identification may increase parental anxiety, while participating families generally consider screening acceptable and perceive its potential benefits to outweigh the associated harms [37]. This distinction between anticipated professional concerns and actual families' reported experiences is important and supports the inclusion of both groups in future evaluations. Acceptability may also vary according to previous experience of T1D, socioeconomic circumstances, cultural background, health literacy, and access to clinical and psychological support.

The development of screening pathways should therefore involve not only healthcare professionals but also children and families. A recent co‐designed study in Western Australia provides an example of how family anxiety, educational needs, psychosocial support, and access to disease‐modifying therapies may be incorporated into the development of clinical pathways for early‐stage T1D [38]. As this study was not part of the evidence included in the present review, it provides contextual evidence rather than confirmation that co‐designed pathways improve implementation outcomes.

This review has several strengths, including a broad search strategy, and the inclusion of quantitative, qualitative, and mixed‐methods evidence. The evidence matrix also allowed findings to be examined according to stakeholder group, screening target, geographical setting, and the implementation context. Most studies were cross‐sectional and relied on self‐reported attitudes or anticipated behaviour or hypothetical perceptions. Potential nonresponse bias and limited sample representativeness were frequent methodological concerns, while routine implementation experience was concentrated in a small number of settings. In addition, the predominance of clinical stakeholders and the limited representation of nonclinical actors constrain the generalisability of the findings to the broader range of stakeholders involved in screening policy, organisation, and implementation.

Further research should move beyond general measures of support and prospectively evaluate professional attitudes, behaviours, and implementation outcomes within clearly defined screening models. Comparative studies should distinguish at‐risk from general‐population screening, primary care from specialist and nonclinical perspectives, and hypothetical from implemented programmes. They should also examine actual resource use, referral and monitoring outcomes, psychosocial effects, equity of access, and the thresholds of clinical benefit, burden, cost, and treatment‐related risk considered acceptable by different stakeholder groups.

## 5 Conclusion

Professional support for early T1D screening in children was conditional rather than uniform and was consistently greater for at‐risk populations than for general‐population screening when the two approaches were directly compared. Professionals recognised potential benefits related to DKA prevention, earlier clinical engagement, and family preparation, but also identified uncertainties regarding psychosocial effects, workforce capacity, costs, and unclear responsibility for communication and follow‐up.

The available evidence primarily reflects the perspectives of clinicians and remains heterogeneous across screening targets, professional groups, healthcare settings, and implementation contexts. Prospective evaluation is needed to determine whether coordinated pathways, professional training, psychosocial support, and sustainable funding models improve the acceptability, feasibility, and outcomes of early T1D screening across different healthcare contexts.

## Author Contributions

F.G. and B.M.M. draughted the initial version of the manuscript based on the study concept and team discussions. O.B. designed and conducted the literature search and serves as the senior author. F.G., B.M.M., A.C., and A.R. contributed to the selection and data extraction processes. F.G., B.M.M., L.M.Q., A.C., G.P., F.M., K.L., S.G., R.D., J.V., A.R., A.M., C.H.d.P., V.E.D.M., K.B., C.M., P.N., and E.B. contributed to the development of the protocol and interpretation of the findings and critically revised the manuscript. All authors reviewed and approved the final version of the manuscript.

## Funding

This work was supported by the Innovative Health Initiative Joint Undertaking (IHI JU) under grant agreement No 101132379 (EDENT1FI). The JU receives support from the European Union's Horizon Europe research and innovation programme, from The Leona M. and Harry B. Helmsley Charitable Trust, from Breakthrough T1D, from EFPIA, from COCIR, from Vaccines Europe, from EuropaBio, and from MedTech. Additional funding is provided to associated UK partners through the UK Research and Innovation (UKRI) Guarantee Fund.

## Conflicts of Interest

P.N. has received advisory board fees from Sanofi and Abbott, speaker fees from Sanofi and Lilly Diabetes, and travel support from Sanofi. R.D. has received funding for advisory board participation, lectures, and talks from Sanofi and Esteve, with all funds paid to the University of Birmingham, and speaker fees from Sandoz. R.D. is supported by a NIHR Personal Award (NIHR304587). A.M. is an employee of Sanofi. C.H.d.P is an employee of BreakthroughT1D. C.M. has received consulting fees from Novo Nordisk, Sanofi, Eli Lilly and Company, Novartis, Dexcom, Boehringer Ingelheim, Bayer, Roche, Abbott, Medtronic, Insulet, Biomea Fusion, SAB Bio, and Vertex; honoraria from Novo Nordisk, Sanofi, Eli Lilly and Company, Dexcom, Boehringer Ingelheim, Bayer, Roche, Abbott, Medtronic, and Vertex; and has participated in data safety monitoring and/or advisory boards for Novo Nordisk, Sanofi, Eli Lilly and Company, Novartis, Dexcom, Boehringer Ingelheim, Bayer, Roche, Abbott, Medtronic, Insulet, Biomea Fusion, SAB Bio, and Vertex. C.M. is President of the EASD and Vice‐President of EUDF. E.B. reported receiving research funding from EDENT1FI, INNODIA and GPPAD European Consortia and Abbott and consulting or lecture fees from Sanofi, Medtronic and Roche. All other authors declare no conflicts of interest.

## Disclaimer

Funded by the European Union, the private members, and those contributing partners of the IHI JU. Views and opinions expressed are, however, those of the author(s) only and do not necessarily reflect those of the aforementioned parties. Neither of the aforementioned parties can be held responsible for them.

## Supporting information


**Table S1:** MEDLINE search strategy.

**Table S2:** Inclusion and exclusion criteria applied during study selection.

**Table S3:** Main characteristics of the studies included in the systematic review.

**Table S4:** Methodological quality assessment of the included studies.

**Table S5:** Summary of the main findings of the included studies across the thematic domains.

## Data Availability

The data that supports the findings of this study are available in the supplementary material of this article.
