# Supplementary material for: Professional Stakeholders' Perspectives on Early Screening for Type 1 Diabetes in Children: A Systematic Review
Source: Diabetes Metab Res Rev. 2026 Oct 19;42(7):e70240. doi: 10.1002/dmrr.70240 (PMC13679756; doi:10.1002/dmrr.70240)
Supplement: Supplementary file 1 [file DMRR-42-e70240-s001.docx]

| **Domain** | **Inclusion Criteria** | **Exclusion Criteria** |
| --- | --- | --- |
| **Population** | Professional stakeholders involved in pediatric T1D care, including clinicians (pediatricians, diabetologists, endocrinologists, physicians, general practitioners), nurses, teachers, health professionals/providers/workers, managers, policymakers, decision-makers, and government representatives. Studies including mixed populations (e.g., professionals and patients/parents) were eligible if professional data were extractable. | Studies exclusively involving patients, parents, or the general public without separately reported professional data. |
| **Exposure / Topic of Interest** | Screening, testing, surveillance, detection, or early diagnosis of Type 1 diabetes (T1D), including references to T1DM, autoimmune diabetes, islet autoantibody testing, insulin-dependent diabetes (IDDM), juvenile diabetes, or diabetes mellitus type 1. | Studies focused exclusively on symptomatic diagnosis, disease management, or treatment without reference to screening or early detection. |
| **Outcomes** | Professional views, perceptions, beliefs, attitudes, evaluations, understanding, knowledge, practices, experiences, decisions, insights, perspectives, or preferences regarding T1D screening. | Studies not reporting professional perspectives or not addressing attitudes or related constructs toward screening. |
| **Study design** | Quantitative, qualitative, and mixed-methods empirical studies. | Reviews, editorials, commentaries, conference abstracts without data and study protocols. |
| **Language** | No restrictions applied |  |
| **Publication status** | Peer-reviewed full-text articles |  |

Supplementary Table 2. Inclusion and exclusion criteria applied during study selection.
