# Supplementary material for: Professional Stakeholders' Perspectives on Early Screening for Type 1 Diabetes in Children: A Systematic Review
Source: Diabetes Metab Res Rev. 2026 Oct 19;42(7):e70240. doi: 10.1002/dmrr.70240 (PMC13679756; doi:10.1002/dmrr.70240)
Supplement: Supplementary file 2 [file DMRR-42-e70240-s003.docx]

| **#** | **Query** |
| --- | --- |
| **1** | Diabetes Mellitus, Type 1/ |
| **2** | ("type* 1 diabet*" OR T1D OR T1DM OR antibod* OR "autoimmune diabet*" OR IDDM OR (insulin dependent adj3 diabet*) OR "juvenil* diabet*").ti,ab. |
| **3** | (screen* OR test* OR surveill* OR detect* OR "early diagnosis").ti,ab. |
| **4** | (stakeholder* OR clinician* OR doctor* OR paediatrician* OR pediatrician* OR diabetologist* OR manager* OR physician* OR teacher* OR nurse*).ti,ab. |
| **5** | government.ti,ab. |
| **6** | general practitioner".ti,ab. |
| **7** | ("policy-maker" OR "policy maker" OR "decision-maker" OR "decision maker").ti,ab. |
| **8** | (health* adj (practitioner OR professional OR worker)).ti,ab. |
| **9** | 1 or 2 |
| **10** | 4 or 5 or 6 or 7 or 8 |
| **11** | (view* OR perception* OR belief* OR attitude* OR evaluation* OR understanding* OR knowledge OR practice* OR experience* OR decision* OR insight* OR perspective*).ti,ab. |
| **12** | 3 and 9 and 10 and 11 |

Supplementary Table 1. MEDLINE search strategy.
